# Adrenal insufficiency presenting as bilateral rigid auricles: a case report

**DOI:** 10.1186/1752-1947-8-302

**Published:** 2014-09-10

**Authors:** Mark Vincent Koning, Ard Struijs

**Affiliations:** 1 Department of Anaesthesiology, Erasmus University Medical Center, Postbus 2040, 3000, CA Rotterdam, The Netherlands; 2 Department of Intensive Care, Erasmus University Medical Center's, Gravendijkwal 230, 3015, CE Rotterdam, The Netherlands

**Keywords:** Adrenal insufficiency, Stiff ears, Petrified auricles, Critical care

## Abstract

**Introduction:**

Stiff ears appear to be a warning sign for adrenal insufficiency. This remarkable and rare sign has not been described to present in adrenal insufficiency in the setting of critical care.

**Case presentation:**

We present the case of a 68-year-old Caucasian male who underwent a thymoma resection and suffered from preoperative weight loss and lack of strength. The perioperative phase was characterised by hypotension and sputum stasis due to muscle weakness, which caused two readmissions to the intensive care unit. His physical examination showed two fully rigid auricles. In retrospect, our patient suffered from secondary adrenal insufficiency and hypogonadism.

**Conclusions:**

The bilateral rigid auricles appeared to be a warning sign for adrenal insufficiency. This remarkable sign is easily checked, and should prompt a higher index of suspicion towards adrenal insufficiency and other hormonal deficiencies.

## Introduction

Stiff ears are a rare clinical sign, especially when the whole auricle is bilaterally affected. A variety of conditions can cause this phenomenon and some of them have important implications for critical care physicians [1].

Here, we present a case report in which stiff ears appeared to be a warning sign for adrenal insufficiency. In this case, preoperative evaluation of the adrenal axis could have optimized our patient prior to surgery and possibly led to an earlier treatment in the postoperative phase.

## Case presentation

A 68-year-old Caucasian male was admitted to the intensive care unit (ICU) for postoperative care after a stage III-A thymoma resection. His history consisted of Ménière’s disease, and a recent onset hypothyroidism which was diagnosed eight months prior by the general practitioner. He received 100mcg levothyroxine once a day, and 8mg betahistine three times a day. The diagnosis of type B1 thymoma was made after an incidental finding of an upper respiratory tract infect on a chest X-ray, of which he recovered fully. Myasthenia gravis was excluded prior to the surgery by negative antibodies against the acetylcholine-receptor, skeletal muscle and muscle specific receptor tyrosine kinase (MuSK). In the months prior to the surgery, he unintentionally lost 15kg and complained of tiredness and muscle weakness. His laboratory studies showed no abnormalities.

The surgery consisted of resection of the thymoma, and the left phrenic nerve and a small section of the left upper lobe were resected due to adhesions. During surgery, he received phenylephrine up to 1.00mcg/kg per minute, after which it changed to norepinephrine up to 0.40mcg/kg per minute. Blood pressure was maintained with a mean arterial pressure (MAP) between 50 and 70mmHg. There was 1240cc of blood loss and 1500cc of Ringer’s lactate, 500cc of Voluven™ (6% hydroxyethyl starch 130/0.4 in 0.9% sodium chloride) and 550 of packed erythrocytes were infused. No corticosteroids were administered.

In the ICU, he suffered from tachycardia and hypotension despite there being little blood loss. His physical examination showed cachexia with decreased muscular strength, and bilateral rigid auricles as the only remarkable sign. The auricles felt calcified and no movement in the auricle was possible. He later told us that this had existed for many years and he could not remember how or when it evolved. There was no history of localized trauma, fight sports, radiation or frostbite. Also, there was no hyperpigmentation. Norepinephrine continued to be administered at 0.4mcg/kg per minute, and additional fluids and 100mg of hydrocortisone were administered to stimulate adrenergic-receptor potency. Serum lactate levels were not elevated, nor was the central venous saturation ever below 70%. During the next day, the norepinephrine was tapered, after which he was discharged to the ward.

On the ward, his MAP persisted between 50 and 60mmHg despite fluid administration. Because the continuous hypotension suggested adrenal insufficiency, hydrocortisone was started at 50mg three times a day and was stopped on the fifth postoperative day (POD). On the seventh POD he was readmitted to the ICU due to respiratory insufficiency based on sputum stasis due to the pre-existing lack of strength. This led to a probable pulmonary infection, although without fever or other signs of infection. His chest X-ray showed left-sided pleural fluid and an atelectasis of the right middle lobe. He was reintubated, inotropics and antibiotics were started and hydrocortisone was restarted at 300mg/day continuously. Later, *Streptococcus agalactiae* was cultured from the sputum. He was extubated three days later and discharged on the eleventh POD to the ward once again.

**Figure 1:**
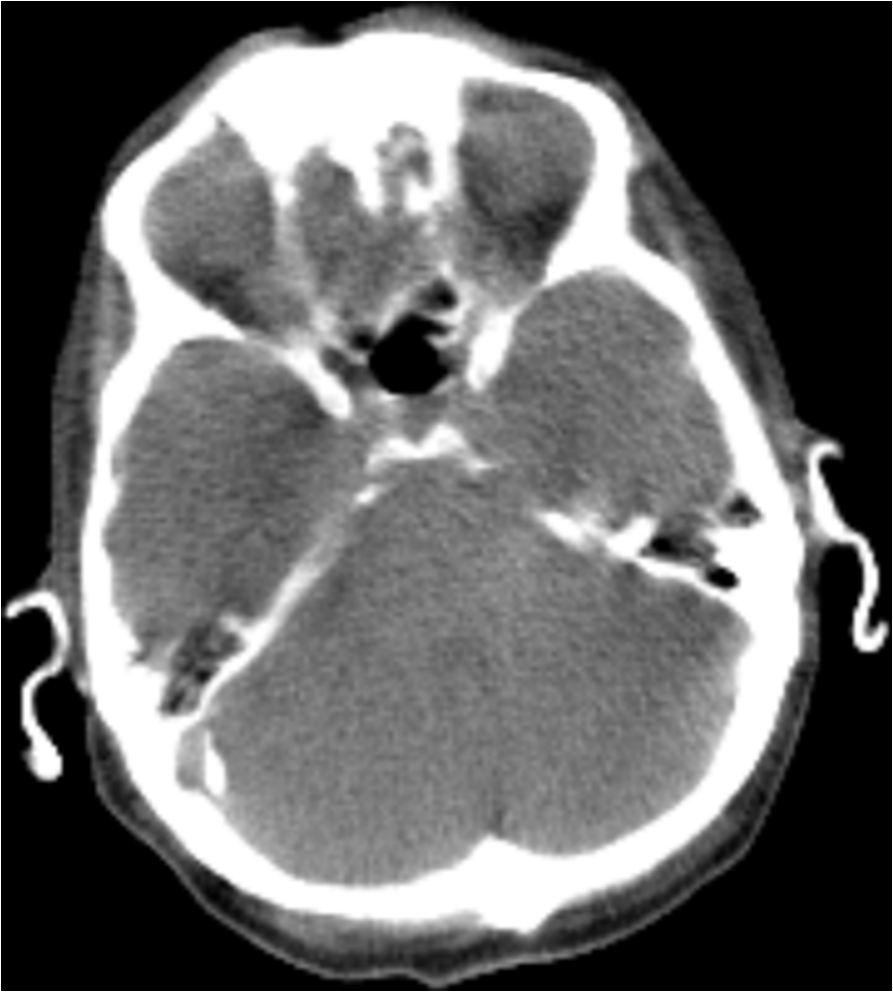
Computed tomography scan of the cerebrum displayed fully calcified auricles.

**Figure 2:**
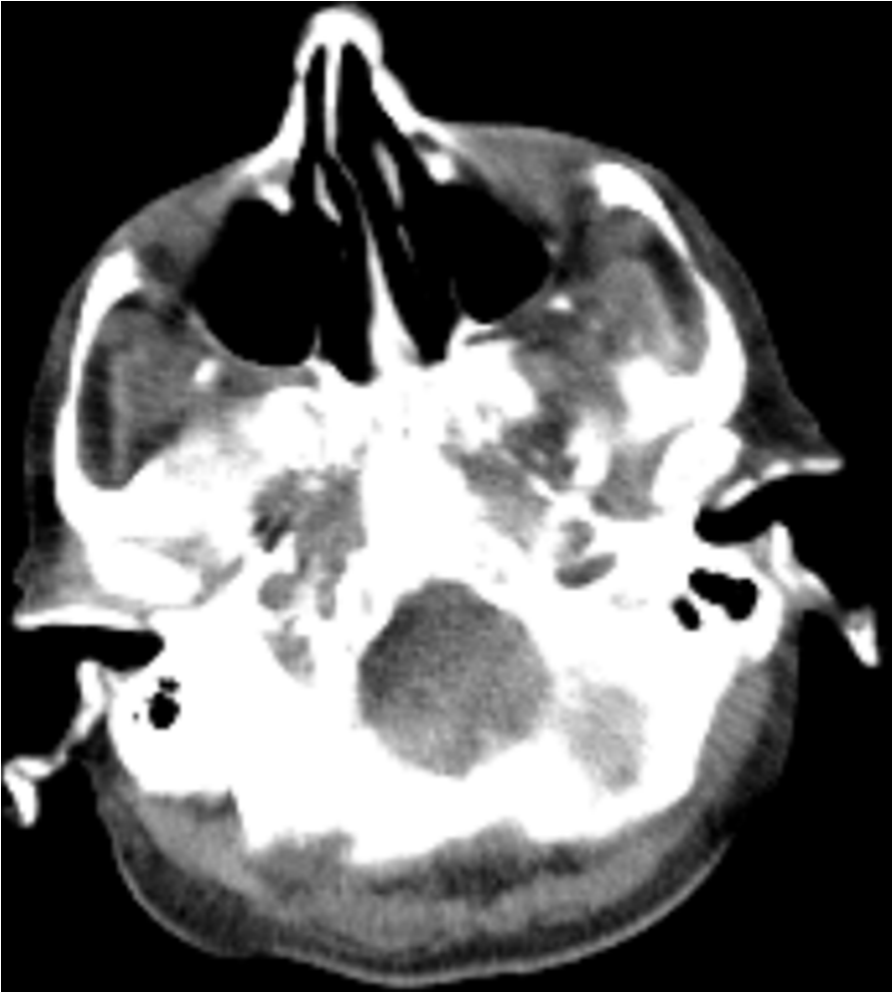
Computed tomography scan of the cerebrum indicates a fully calcified external auditory canal.

On the 14th POD he was re-admitted to the ICU for the second time due to respiratory insufficiency based on diminished airway secretion clearance, while receiving 50mg hydrocortisone once a day. He was re-intubated and a tracheostomy was performed. Hydrocortisone was continued at 75mg/day and he was quickly weaned off the ventilator. He was discharged to the ward on the 18th POD.He was in a stable phase on the 21th POD, so the pituitary axes were evaluated. His laboratory tests showed: cortisol 51nmol/L (normal 200 to 700nmol/L), TSH (thyroid-stimulating hormone 11.9mU/L (n=0.4 to 4.3mU/L), free T4 11.2pmol/L (n= 11 to 25pmol/L), lutein hormone (LH) 6.5U/L (n=1.5 to 8.5U/L), FSH (follicle-stimulating hormone) 10.9U/L (n=2 to 7U/L), testosterone radioimmunoassay 3.4nmol/L (n=10 to 30nmol/L), prolactin 0.49U/L (n=0-0.36U/L), and IGF-1 (insulin-like growth factor 1) 7.8nmol/L (n=6 to 25.9nmol/L). A magnetic resonance imaging scan of his cerebrum showed pituitary hypoplasia. A re-evaluation of his preoperative computed tomography (CT) scan showed bilateral totally calcified auricles (Figures 1 and 2), however no other signs of calcifications were found. The adrenal glands appeared normal. A metyrapone test was performed in an out-patient setting and displayed secondary adrenal insufficiency.

## Discussion

Calcification of the auricles is described in many forms and multiple terms are used for this heterogeneous curiosity, such as ‘petrified’, ‘rigid’, ‘stiff’, ‘calcificated’ and ‘ossificated’ [2]. In this case both the auricles and the external auditory canal were calcified as presented in Figures 1 and 2. The ears appeared normal externally, slightly more pale than the facial color. They were fully rigid, and no manual folding movement was possible. No histology was obtained, therefore we cannot comment on the distinction between calcification and ossification.

The incidence of a fully rigid auricle is low; Scherrer examined 800 patients and found no auricle rigidity [3]. However, Gossner found calcification of the auricle in 19.5% of CT scans of the cerebrum, indicated by a variety of reasons (e.g. stroke, malignancies, trauma, dementia) in 2013, but it is unknown if he found any auricle rigidity [4]. We would like to stress that calcifications are not the same as auricle rigidity. Calcified noduli in the auricle is classified as calcification of the auricle as well, but it will not give auricle rigidity. Frost bite, trauma, hypercalcemia, inflammation and radiotherapy are described as causes of calcificated noduli and rigid ears, both bilateral as unilateral. However, cases of adrenal insufficiency are only associated with bilateral rigid ears [5].

Stiff ears are usually asymptomatic and give very little burden. However, when patients report themselves, it is usually in an out-patient setting and due to secondary problems (such as troubles with a hearing aid) [6]. Thus far, two cases involved critical care medicine, making it even rarer for clinicians in this specialty [7,8]. While those two reports were medical emergencies, this case is a scheduled surgical patient, which leaves room for preoperative optimization or earlier recognition of adrenal insufficiency.

When associated with hormonal disturbances, the etiology of stiff ears is far from elucidated and several theories have been postulated [8-10]. Since this sign has been reported in both primary and secondary adrenal insufficiency, cortisol deficiency is likely to play a role, regardless of the calcium and phosphorus levels [9]. Furthermore, the rigid ears and the association of endocrinological disorders is only reported in male patients, therefore gonadal hormones could be involved [5].

Preoperatively, the hormonal status should be evaluated in patients with suggestions of hormone deficiency. In this case the weight loss, lack of strength, prediagnosed hypothyroidism and bilateral rigid ears would justify hormone axis evaluation. A common hypothesis of weight loss due to the thymoma which than leads to a lack of strength sounds reasonable, however a thymoma is seldom accompanied by weight loss [11].

To test the hypothalamic-pituitary-adrenal (HPA) axis one should measure the serum free cortisol level between 8 and 9 am. A free cortisol level under 83nmol/L could indicate hypopituitarism and should prompt for further testing. When cortisol levels are over 497nmol/L, it indicates that basal ACTH (adrenocorticotropic hormone) secretion is sufficient. In hypopituitary patients the thyroid status is determined by measuring thyroxine or free T4, since TSH levels are usually normal. The gonadal axis in male patients is evaluated by determining the testosterone and LH. A serum IGF-1 concentration indicates growth hormone deficiency when lower than the age-specific lower limit [12].

In the critically ill, the diagnosis of adrenal insufficiency is much debated. Cortisol concentrations do not reflect adrenal function and are affected by hypoproteinemia [13]. The synthetic ACTH test is less reliable and at risk of showing a false positive in the postoperative phase, as exemplified by Debono *et al.*[14,15]. Therefore, it is recommended to treat the patient with glucocorticoids when adrenal insufficiency is clinically suspected [15]. Hydrocortisone up to 200mg a day is the preferred corticosteroid [16].

Myasthenia gravis (MG) needed to be excluded in our patient who suffered from decreased sputum clearance and a thymoma, which is frequently accompanied with MG [17]. Virtually all patients with a thymoma and clinical signs are sero-positive for ACH-r antibodies, so a negative test would exclude MG [18]. We tested our patient again for antibodies during the ICU admission, and obtained similar negative results as those from his preoperative test. Furthermore, he exhibited no typical signs for MG.

In retrospect, the initial TSH (13.8mU/L) and FT4 (8.3pmol/L) levels were requested by the primary caregiver, which showed a primary hypothyroidism. As previously mentioned, his weight loss prior to the surgery is unlikely to be explained by the thymoma, but is associated with endocrinological disorders such as hypothyroidism and hypopituitarism [11]. His testosterone deficiency could be attributed to the pre-existing lack of muscular force, which also contributed to the sputum stasis. His HPA axis tests afterwards indicated secondary adrenal insufficiency. We hypothesize that early initiation of gonadal and adrenal hormone substitution could lead to a less complicated postoperative course.

We would like to draw attention to this rare clinical sign and its use as an indicator either primary or secondary adrenal insufficiency. Especially in conjunction with other signs of adrenal insufficiency, ‘stiff ears’ could lead to a higher index of suspicion, yield an improved treatment course and likely less morbidity.

## Conclusions

We presented the case of a patient with bilateral rigid ears, who suffered from adrenal insufficiency and hypogonadism. These rigid ears have been associated with adrenal insufficiency, although no clear etiology is known. This remarkable sign is easily checked, and should prompt a higher index of suspicion towards adrenal insufficiency. We hypothesize that early hormonal substitution could have led to a less complicated postoperative phase.

## Consent

Written informed consent was obtained from the patient for publication of this case report and any accompanying images. A copy of the written consent is available for review by the Editor-in-Chief of this journal.

## Abbreviations

ACTH: Adrenocorticotropic hormone; ACH-r: Acetylcholine-receptor; FSH: Follicle stimulating hormone; HPA: Hypothalamic–pituitary–adrenal; ICU: Intensive care unit; IGF: Insulin-like growth factor; LH: Luteinizing hormone; MAP: Mean arterial pressure; MG: Myasthenia gravis; MuSK: Muscle-specific receptor tyrosine kinase; POD: Postoperative day; TSH: Thyroid stimulating hormone.

## Competing interests

The authors declare that they have no competing interests.

## Authors’ contributions

Both authors were involved in the treatment of the patient and contributed equally on the writing of the manuscript. Both authors read and approved the final manuscript.
